# Association between Cardiometabolic risk factor and responsiveness to vitamin D supplementation: a new approach using artificial neural network analysis

**DOI:** 10.1186/s40795-021-00413-7

**Published:** 2021-04-08

**Authors:** Elahe Allahyari, Parichehr Hanachi, Seyed Jamal Mirmoosavi, Gordon A.Ferns, Afsane Bahrami, Majid Ghayour-Mobarhan

**Affiliations:** 1 Department of Epidemiology and Biostatistics, School of Health, Social Determinants of Health Research Center, Birjand University of Medical Sciences, Birjand, Iran; 2 Department of Biology, Biochemistry Unit, Alzahra University, Tehran, Iran; 3 Community Medicine, Community Medicine Department, Medical School, Sabzevar University of Medical Sciences, Sabzevar, Iran; 4 Division of Medical Education, Brighton & Sussex Medical School, Falmer, Brighton, Sussex, BN1 9PH UK; 5 Cellular and Molecular Research Center, Birjand University of Medical Sciences, Birjand, Iran; 6 Metabolic Syndrome Research Center, School of Medicine, Mashhad University of Medical Sciences, Mashhad, Iran

**Keywords:** Waist to hip ratio, Adolescent girls, Artificial neural network, Waist circumference

## Abstract

**Background:**

There are increasing data highlighting the effectiveness of vitamin D supplementation in the treatment of vitamin D deficiency. But individuals vary in their responsiveness to vitamin D supplementation. In this study, the association between several cardiometabolic risk factors and the magnitude of response to vitamin D supplementation (change in vitamin D level) was investigated using a novel artificial neural networks (ANNs) approach.

**Methods:**

Six hundred eight participants aged between 12 to 19 years old were recruited to this prospective interventional study. Nine vitamin D capsules containing 50,000 IU vitamin D/weekly were given to all participants over the 9 week period. The change in serum 25(OH) D level was calculated as the difference between post-supplementation and basal levels. Suitable ANNs model were selected between different algorithms in the hidden and output layers and different numbers of neurons in the hidden layer. The major determinants for predicting the response to vitamin D supplementation were identified.

**Results:**

The sigmoid in both the hidden and output layers with 4 hidden neurons had acceptable sensitivity, specificity and accuracy, assessed as the area under the ROC curve, was determined in our study. Baseline serum vitamin D (30.4%), waist to hip ratio (10.5%), BMI (10.5%), systolic blood pressure (8%), heart rate (6.4%), and waist circumference (6.1%) were the most important factors in predicting the response to serum vitamin D levels.

**Conclusion:**

We provide the first attempt to relate anthropometric specific recommendations to attain serum vitamin D targets. With the exception of cardiometabolic risk factors, the relative importance of other factors and the mechanisms by which these factors may affect the response requires further analysis in future studies (Trial registration: IRCT201509047117N7; 2015-11-25; Retrospectively registered).

## Background

Vitamin D (VitD) is a fat soluble vitamin, the major source being dermal synthesis under ultraviolet light exposure, although it is also obtained in limited amounts from specific food intake [1]. VitD deficiency appears to contribute to the development of several chronic diseases [2–4]. In recent years, the prevalence of VitD deficiency has increased among healthy children and adolescents globally [5–7]. In observational studies, several factors have been found to be related to a low VitD status, for example female gender, elderly, low socioeconomic status, high latitude of residence, non-white ethnicity, overweight, less outside physical activity, and dietary intake [8–10].

There are accumulating data highlighting the importance of supplementation as an effective approach to solve the problem of VitD deficiency [11–14]. Preservation of VitD stores without supplementation or careful attention to dietary resources is difficult, particularly in environments where sun exposure and natural food source is restricted [15]. Furthermore individual responses to supplementation are variable. VitD type and dose, baseline VitD level, age, season, latitude, simultaneous use of calcium supplement, and body mass index (BMI) are parameters that have been shown to be affect responsiveness to VitD supplementation [16–21].

The prevalence of overweight among adolescents has manifested an alarming increase, which absolutely reach a pinnacle in adults more severe obese [22, 23]. Obesity is one of the main public health problems around the world. Central obesity is related to metabolic syndrome (MetS), insulin resistance, type 2 diabetes mellitus and atherosclerotic cardiovascular disease (CVD) [24]. Due to the negative association between VitD concentrations and the degree of obesity, and central adiposity [25], a potential role of hypovitaminosis D in the pathogenesis of the MetS has also been proposed [26]. There are numerous parameters that predict overweight/obesity, cardiovascular risk factors, and disease. There are some simple methods that can be used to evaluate these parameters in primary care facilities, i.e. measurements of body weight (BW), height, neck circumference (NC), waist circumference (WC), wrist circumference, hip circumference (HC), blood pressure (BP) and calculations of waist-to-hip ratio (WHR) and BMI.

Despite the considerable cross-sectional interrelations of low serum (S)-25(OH) D levels with the components of MetS of which have cardiovascular effects, there are no data on whether anthropometric parameters can modify the magnitude of the effect of VitD supplementation to correct VitD deficiency.

Previous studies have analyzed their data using canonical statistical methods that are based on the presumptions of linear relationships between variables. These approaches have less statistical power in the assessment of non-linear and complex relationships, as commonly detected in biological pathways. Recently, these limitations have been overcome through the application of a novel data mining analysis, artificial neural network (ANN) [27]. The ANNs similar to other machine learning algorithms were formerly exerted in various studies to recognize predictive factors of different chronic diseases [28, 29].

In this study we have evaluated the association between cardiometabolic risk factors and the increments in serum 25(OH) D levels in response to very high dose VitD supplements in a large sample of adolescents girl by using ANN approach.

## Methods

### Study design and populations

This prospective and interventional study was performed in January–April 2015 at Mashhad and Sabzevar cities, Iran, as described previously [7, 30]. Eligible subjects (*n* = 640) received a 50,000 IU soft-gel capsule VitD/weekly for 9 weeks, according to guidelines [31]. The Ethics Committee of our University (MUMS) approved all the study protocol, and written informed consent was signed by all participants and their guardians.

Physical activity was estimated by a validated questionnaire and reported as metabolic equivalents (METs) in hours/daily [32]. Demographic data and use of sunscreen were gathered via an expert interview. Regarding passive smoking status, all participants in the study were instructed to respond to the question [33]: “Do one/or both of your guardians currently smoke cigarettes/tobacco and are you exposed to smoke > 1 h/daily?

### Anthropometric and cardiac measurements

Anthropometric parameters including BW, height, NC, WC, HC, Heart rate (HR), wrist circumference, systolic BP (SBP) and diastolic BP (DBP) were measured in duplicate based on the standard NHANES III procedure as described previously [7], and then BMI and WHR was computed. When the first two measures varied even 0.3 cm, a 3rd measurement was undertaken, and the mean of all measures recorded was calculated.

### Blood collection and VitD assessments

Fasting blood samples were obtained early in the morning at baseline and after 9 weeks’ trial. An electrochemiluminescence (ECL) technique (Roche, Basel, Switzerland) was recruited for the measurement of S-25(OH)D.

### Statistical analysis

Normality of data was checked using the Kolmogorov-Smirnov test. Frequency or mean ± standard deviation (SD) reported descriptive statistics. Then, sociodemographic and cardiometabolic risk factors were compared by Kruskal-Wallis/one-way ANOVA, or chi-square/Fischer’s exact test in the different tertiles of increments in serum 25(OH) D in response to supplementation. *P* value < 0.05 was set as significance.

#### ANN system for predicting VitD response to supplementation

ANN technique approximate relationship function between input and output data by nonlinear processing elements (named neurons) that are connected in a parallel structure like biological neurons in the human brain [34]. The ANN model with one hidden layer can efficiently approximate any continuous variable when number of hidden neurons are sufficient [35]. However, there is a lack of consensus surrounding the general rules to find optimal number of hidden neurons and transfer functions between layers [36]. Furthermore, the feed forward network is one of the most widely used ANNs designed for model processing, forecasting, pattern discrimination and classification [37].

The data were randomly divided into two groups (70% for training and 30% for testing set) by using Statistical 100 Package for Social Sciences version 19 (SPSS Inc., Chicago, Illinois, USA). Then, the feed-forward ANN with back-propagation algorithm were used to train data with different algorithms (hyperbolic tangent or sigmoid transfer function in hidden layers and linear, softmax, hyperbolic tangent or sigmoid transfer function in output layer) and different number of neurons (between 2 and 50) in one hidden layer model. Therefore, factors of the ANN model including Initial Lambda, Initial Sigma, Interval Center, Interval Offset, and Maximum Training Epochs were 5e-7, 5e-5, 0, ±0.5, and automatically, respectively. The input variables used were: age, use of sun protective cream (no, yes), passive smoker status (no, yes), baseline serum VitD level, BMI, physical activity, heart rate, SBP and DBP, NC, WHR, wrist circumference, HC, and WC. Finally, the optimum ANN structure was selected according to obtained mean sum of square errors of three replicate in both training and test sets. After that, according to 33th and 66th percentile of ∆25(OH) D level or the difference between post-supplementation and basal levels, output layer is categorized like: low (< 20.80 ng/mL), moderate (between 20.81 to 34.57 ng/mL), and high (> 34.57 ng/mL) and performance of selected ANN architecture was expressed according to the sensitivity, specificity, and accuracy area under the ROC curve (AUC).

## Result

All 640 subjects received capsules of 50,000 IU of VitD, and 620 completed the 9 weeks supplementation. In present study, full data were available for 608 adolescent student girls aged between 12 to 19 years old. At baseline, 74.5, 15.5 and 10% of participants suffered from severe VitD deficiency (< 10 ng/ml), VitD deficiency (< 20 ng/ml) and VitD insufficiency (< 30 ng/ml). Serum levels of 25(OH) D (9.4 ± 8.8 vs., 36.4 ± 15.6 ng/mL; *P*-value < 0.001) were elevated significantly by the end of study versus the baseline. The mean net change in 25(OH) D post intervention was 26.9 ± 16.4 ng/ml.

Table 1 reports comparison of demographic and cardiometabolic risk factors between the tertiles of response to serum VitD categories. As the table clearly shows, only baseline serum 25(OH) D, age, WHR, and WC had significant difference between three tertile groups of response to supplementation (*P* < 0.05).

**Table 1:** Comparison of demographic and cardiometabolic risk factor between the tertiles of response to vitamin D categories

Variables		Response to treatment			*P* value
		1st Tertile (*n* = 193) ≤20.80 ng/mL	2nd Tertile (*n* = 212) 20.81–34.58 ng/mL	3rd Tertile (*n* = 203) > 34.58 ng/mL	
Basal 25(OH) D (ng/mL)		13.7 ± 12.7	7.7 ± 5.7	7.37 ± 4. 8	**< 0.001**
Age (year)		14.9 ± 1.5	14.5 ± 1.5	14. 6 ± 1.5	**0.01**
Physical activity (MET/h)		45.8 ± 4.0	44.9 ± 2.8	45.5 ± 3.8	0.07
BMI (kg/m2)		21.4 ± 3.9	21.2 ± 4.3	20.5 ± 3.7	0.11
Use of sun protective cream	Yes	84 (31.8)	93 (35.2)	87 (33.0)	0.98
	No	109 (31.7)	119 (34.6)	116 (33.7)	
Passive smoking exposure	Yes	125 (31.1)	139 (34.6)	138 (34.3)	0.78
	No	68 (33.0)	73 (35.4)	65 (31.6)	
Heart rate		83.5 ± 13.4	82.5 ± 12. 7	83.7 ± 12.9	0.60
SBP (mm Hg)		96.9 ± 14.7	96.7 ± 15.8	95.7 ± 13.5	0.65
DBP (mm Hg)		62.9 ± 14.8	61.2 ± 13.5	63.1 ± 11.4	0.14
NC (cm)		31.1 ± 2.4	31.40 ± 2.3	30.9 ± 2.1	0.10
WHR		0.76 ± 0.06	0.78 ± 0.06	0.76 ± 0.06	**0.005**
Wrist circumference (cm)		15.1 ± 1.16	15.2 ± 1.05	15.1 ± 1.01	0.31
HC (cm)		92.4 ± 8.70	91.1 ± 9.14	90.8 ± 8.7	0.15
WC (cm)		70.5 ± 8.58	70.7 ± 9.02	68.9 ± 8.3	**0.04**

Data presented as mean ± SD or number (%). *P*-value is obtained by Kruskal Wallis Test (non-normally distributed variables) or Chi-square test (categorical variables). Significant of bold value are< 0.05 levels. *Abbreviation*: *SBP* systolic blood pressure, *DBP* diastolic blood pressure, *NC* neck circumference, *WHR* waist-to-hip ratio, *WC* waist circumference, *HC* hip circumference

**Fig. 1:**
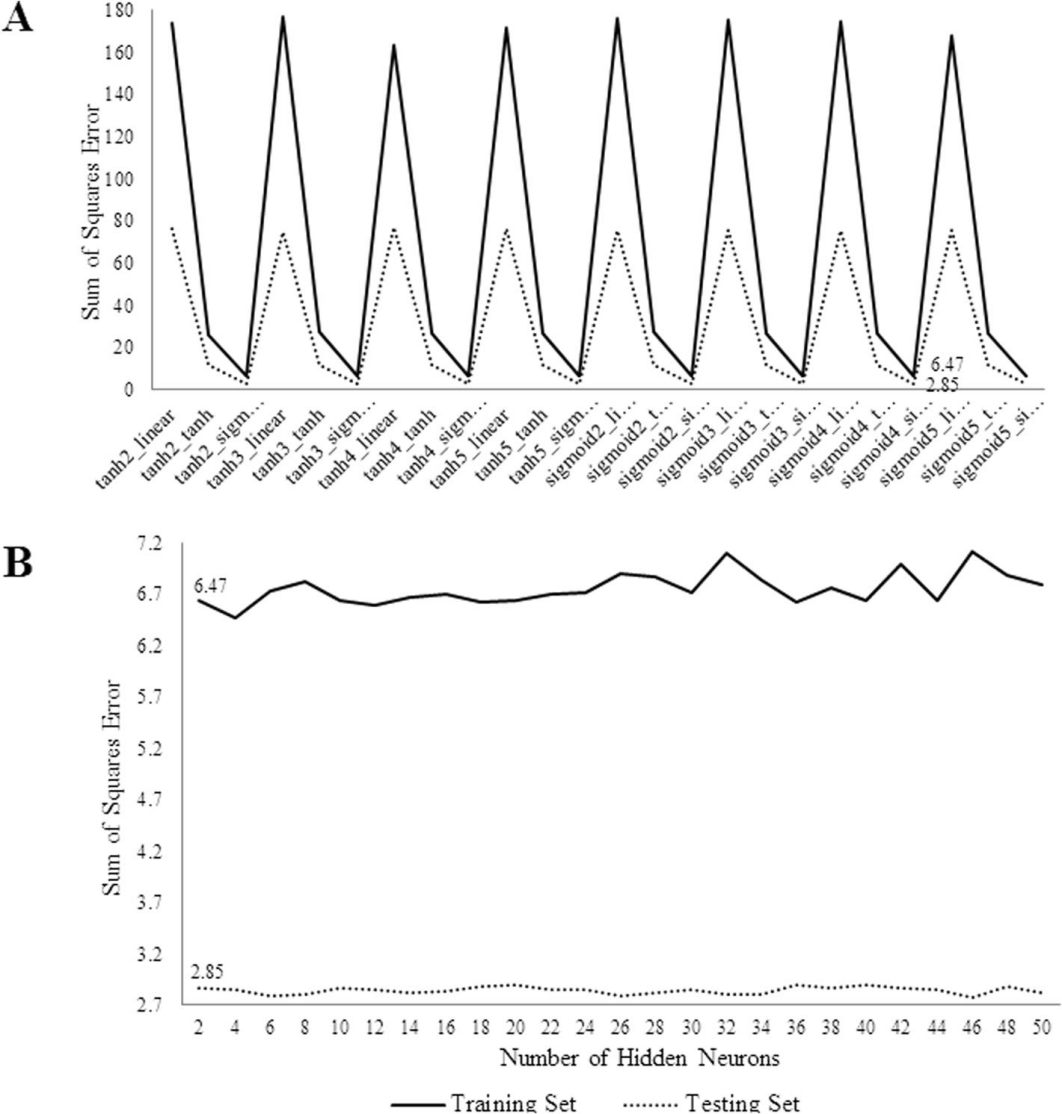
**a** The overall sum of squares error of ANN models for different transfer functions; **b** The sum of squares error of selected ANN transfer function models for different hidden neurons

We obtained sum of square error of ANNs algorithm. We selected suitable transfer function in hidden and output layers (Fig. 1a) and sufficient number of hidden neurons (Fig. 1b). With increasing hidden neurons, over fitting in ANNs algorithms were prevented when sum of square error in training and test sets were closed. Figure 1a revealed that a sigmoid function at both hidden and output layers had the best performance with 6.47 and 2.85 sum of square error in training and testing sets.

The best performance was also found using the ANN algorithm with 4 hidden neurons in Fig. 1b.

**Fig. 2:**
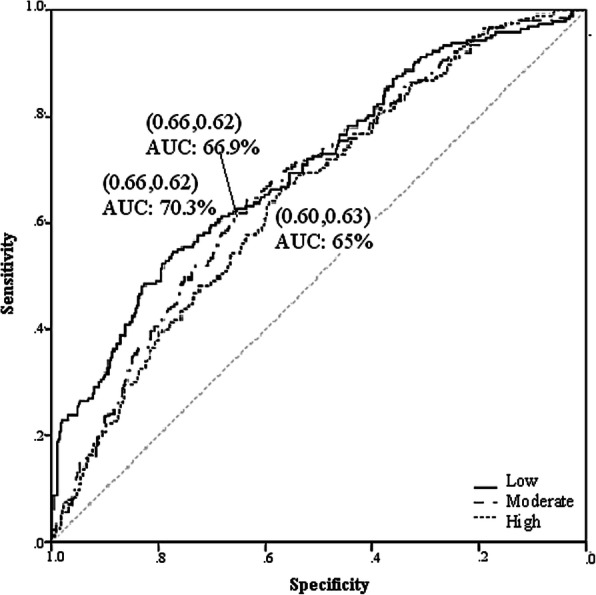
Roc curve of the Artificial Neural Network model

**Fig. 3:**
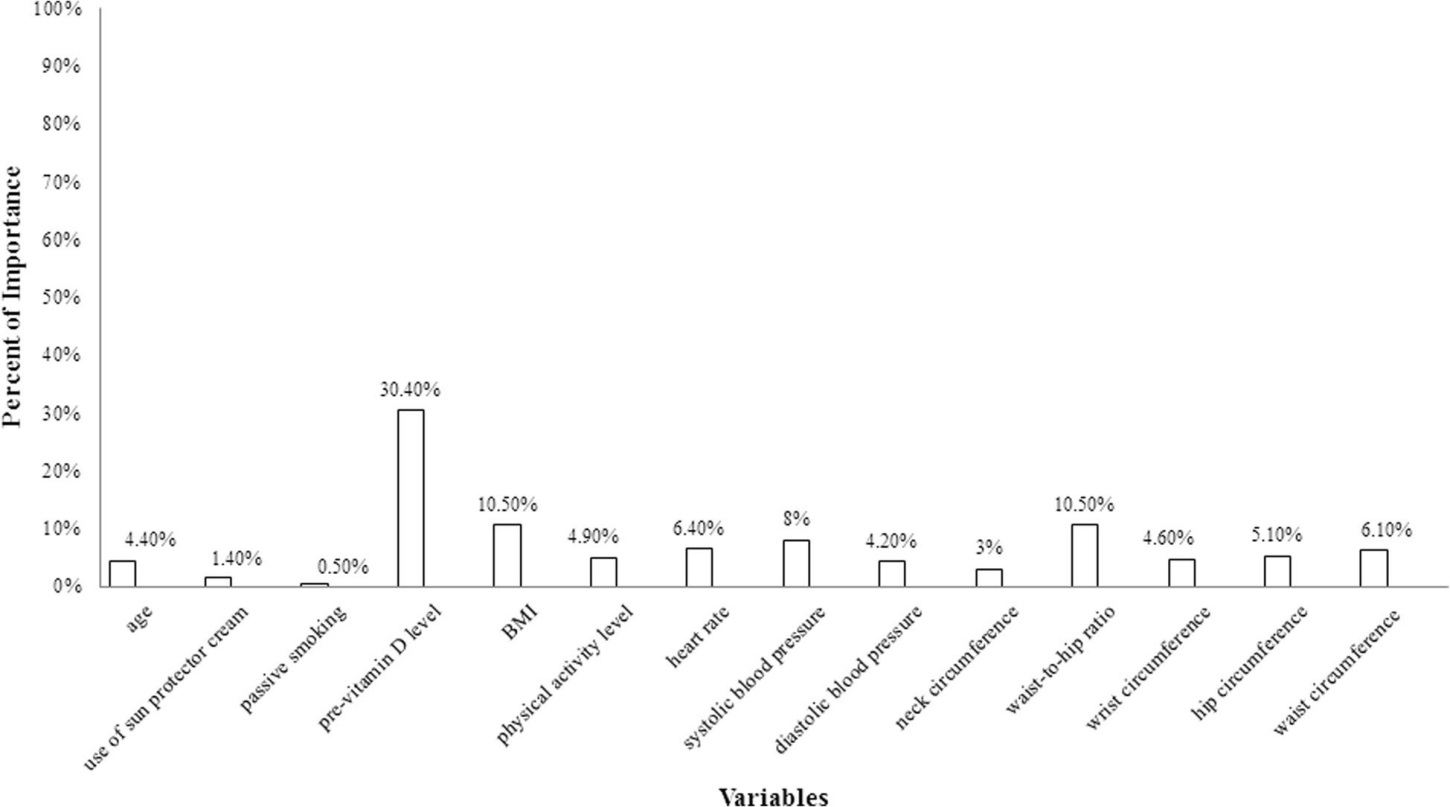
The variable importance from the selected Artificial Neural Network

Figure 2 shows the specificity, sensitivity, and AUC values of 66, 62, 70.3% for low, 66, 62, 66.9% for moderate, and 60, 63, 65% for high responsiveness to VitD supplementation respectively. The variable importance in Fig. 3 displayed that the top-6 determinants of ∆25(OH) D were basal VitD (30.4%), WHR (10.5%), BMI (10.5%), SBP (8%), WC (7.2%), HR (6.4%), and WC (6.1%). Other factors had < 6% importance. Therefore, cardiometabolic variables were found to be the most important predictive factors after baseline serum VitD.

## Discussion

Obtaining VitD from food sources whilst ideal, is usually difficult to achieve because of its low concentrations in unfortified foods. Daily supplementation may be appropriate to preserve a constant serum level of 25(OH) D, though compliance with a daily regimen can be a big challenge in some cases (58). Taking a high-dose of VitD3 (50,000 IU per week) is recommended for hypovitaminosis D therapy [38], so in the present trial, we administrated 9 high-dose VitD capsules (50,000 IU cholecalciferol per week). To identify novel and hidden determinants that explain the individual variations in the response to the VitD supplementation, we applied a best data mining model. Results of the present study highlight the importance of cardiometabolic risk factors in characterization of response to VitD intervention.

In adolescents girls we found that basal serum levels and age determined the increment in serum 25(OH) D following the use of supplements. Rahmanian et al. reported that baseline serum VitD amounts and geographical region are determinants of the magnitude of responsiveness to supplementation [39]. Recently, a systematic review and meta-analysis of randomized controlled trials (RCTs) revealed that baseline 25(OH) D concentration and age were significant determinants of changes in 25(OH) D concentration following VitD treatment [40]. The inverse association between baseline levels of S-25(OH) D and changes in 25(OH) D in response to VitD intervention may be due to the a negative feedback of 25-hydroxylase activity [41]. Aging has commonly reported to be related with decreased circulating values of 25(OH) D [42, 43]. Although, other evidence has reported that aging has little or no effect on response to supplementation [44–47]. The reason for the contradictory findings is the mean age of the volunteers is different between studies.

With our model, the 29.3% increase in serum 25(OH) D following VitD supplementation can be predicted if anthropometric parameters including WHR, NC, WC, wrist circumference, and HC levels are known.

There is accumulating evidence of an inverse relationship between serum 25(OH)-D and indices of adiposity, including weight, BMI, percent body fat, WC and WHR. For instance, the BW, BMI, and WC of the women with ≥90 nmol/l serum 25(OH) D were significantly lower compared to women with < 90 nmol/l serum 25(OH) D subjects. But, the HC and the WHR were not differed between both groups [48]. Furthermore, Tamer and co-researchers found that serum 25(OH) D levels were inversely associated with BMI, WC and WHR (r = − 0.48, *p* < 0.0001; r = − 0.48, p < 0.0001 and r = − 0.31, *p* < 0.05, respectively). The authors concluded that hypovitaminosis D in lacking of diabetes type 1 and hyperparathyroidism may be associated with obesity/abdominal obesity [49]. Similarly Vilarrasa et al by using bivariate correlation analysis reported that the serum 25(OH) D levels were inversely correlated with BMI (r = − 0.43, *p* = 0.001) and WHR (r = − 0.40, *p* = 0.001) [50]. In a population-based study in elderly, higher BMI, and WC were significantly related with lower serum 25(OH) D (standardized β values = − 0.136, and − 0.137, respectively; *P* < 0.05), after adjustment for possible confounders [51].

One of the explanation for the relationship between obesity and lower VitD levels is because of the higher capacity of VitD storage in the fat tissue or the interaction with autocrine elements generated via adipose tissues [52, 53]. But, the reverse of causal inference of higher BMI in the attenuating of VitD status was not proven [54]. It is also suggested that the higher serum 25(OH) D conversion to 1,25(OH)2D found in obese cases [55] may be increased in obese individuals with low serum 25(OH) D concentrations versus obese subjects with higher concentrations causing to great 25(OH) D consumption.

In agreement with our findings, results from previous studies using classical linear statistical methods highlighted the hallmark of anthropometric indices in variance of S-25(OH)-D levels post-supplementation. Previous reports from particular age groups highlighted a significant role for BW for prediction of variation in 25(OH) D levels after intervention [56–58] even compared to body fat mass [56]. Blum and co-workers reported that magnitude of increasing in serum value of 25(OH) D concentration in response to supplementation negatively related with BW, BMI, central body fat, and waist round in elderly [59]. Twelve-week VitD supplementation in healthy overweight and obese female led to statistically significant reduction in body fat mass compared to the placebo group, however, BW and WC did not change significantly in intervention and placebo groups. A significant inverse association between variations in serum 25(OH) levels and body fat mass was found (r = − 0.319, *P* = 0.005) [60].

WC and WHR are the most prevalent representative measures of visceral adipose tissue. But, WHR may be a superior predictor of CVD risk as HC is inversely related with the evolution of cardio-metabolic risk factors [61–63]. An interesting and novel finding of our algorithm is the independent, relationship of VitD responsiveness with WC and WHR. Pasco and colleagues observed that women with a normal WC were 1.5–fold more likely compared to women with a higher WC to have high serum 25(OH) D (OR = 1.46, 95% CI:1.02–20.8; *p* = 0.038) [64].

NC was identified as the third significant predictor that independently affected the response of S-25(OH) D to VitD supplementation in current study. NC, as an indicator of upper body subcutaneous fat distribution suggested having potential for using as identification of overweight/obese individuals. From the anatomical standpoint, upper-body subcutaneous adipose tissue is a unique fat storage situated in a separate section compared with visceral adipose tissue. Systemic free fatty acid levels are mainly derived from upper-body subcutaneous fat, indicating that this fat storage may be involved in the risk of CVD [65, 66]. NC as a measure of neck fat is a very simple, convenient and reliable alternative measure of obesity and may even be an better independent marker of metabolic risk versus BMI and WC [67, 68].

Wrist circumference measurement being easy-to-detect, and noninvasive may be a good surrogate to analyze bone metabolism because it is an simple to measure the skeletal frame without being significantly confounded via variation in body fat and perturbing factors [69].

Unlike other anthropometrics, it has a high reproducibility because it does not need multiple repeated assessments for precision and reliability [70]. Wrist circumference could be proposed as a novel anthropometric measurement for prediction of insulin resistance, metabolic syndrome and CVD [71]. But it could only explain 4.6% of total slope representing the elevation in S-25(OH) D concentration post VitD therapy.

The renin–angiotensin system (RAS) contributed in the regulation of BP, volume and electrolyte homeostasis. Dys-activation of the RAS may cause to hypertension. VitD is an effective endocrine suppressor of renin production and a negative regulator of the RAS. In animal model, lacking the VitD receptor (VDR) has elevated production of renin and angiotensin II, leading to hypertension. Low VitD status has been connected with an higher risk of cardiovascular disease and [72, 73] hypertension [74]. Vimaleswaran and co-researchers reported that elevated 25(OH) D values were related with lower SBP (β per 10% alteration = − 0.12 mmHg, 95% CI:–0.20 to − 0.04; *p* = 0.003) and decreased risk of hypertension (OR = 0.98, 95% CI:0.97–0.99; *p* = 0.0003); but, they did not found an relationship between 25(OH) D level and DBP (β = − 0.02 mmHg, 95% CI:–0.08 to 0.03; p = 0·37) [75]. VitD can suppress renin biosynthesis with influencing the juxtaglomerular apparatus [76] and actually endothelial cells contain VDR, so offering a favorite vascular substrate for VitD to perform actions [77]. Moreover, VitD can repress parathyroid hormone production, itself related with CVD, and can inhibit generation of pro-inflammatory cytokine [78], which has been contributed in the enhancement of arterial stiffness. In a meta-analysis including 46 trials with 4541 subjects, no effect of VitD supplementation was found on SBP and/or DBP [79]. However, we could demonstrate that SBP and DBP is a modifier of 12.2% of incremental 25(OH) D in individuals on VitD supplements.

Moreover, responsiveness to VitD treatment is a multifactorial condition in which various parameters interact in non-linear biological pathways, which likely require a particular mathematical method, i.e. ANNs, to be understood. It has been suggested that ANN analysis offers a promising alternative to traditional statistical techniques for the statistical analysis of multivariate data in order to finding patterns in data encompassing many variables [80]. In current study, the feed-forward ANN with back-propagation as the training algorithm has been used to computing the magnitude of response to supplementation concerning to cardiometabolic risk factors in large population. But this research was limited solely to the adolescent girls population. Regarding to the ethical consideration, we were not able to have a control group in the current study which is another of our limitation. Furthermore, the study was performed in January–April (from winter to spring). There is a significant increase in VitD at this time of year even without intervention. So, it is unclear whether the change in VitD during intervention was solely due to the supplementation or also because of seasonal exposure to sunshine or other factors. Therefore, it is required to interpret our data cautionally.

## Conclusion

Our findings are the first to relate anthropometric specific recommendations to reach serum 25(OH) D targets. The main predictors of increments in serum 25(OH) D concentration in response to supplement use were baseline VitD levels, WHR, BMI, SBP, HR, and WC respectively. However, prediction of Vit D response remains an open issue. Future studies are required to confirm these results and evaluated other plausible factors may be related to response to supplementation such as genetic factors, VitD type and dose as well as overall physical and psychological status in order to application to prevent VitD deficiency strategies in the general population.

## Data Availability

The datasets used and/or analyzed during the current study are available from the corresponding author on reasonable request.

## Abbreviations

ANNs: Artificial neural networks
AUC: Area under the ROC curve
BP: Blood pressure
BW: Body weight
CVD: Cardiovascular disease
DBP: Diastolic blood pressure
ECL: Electrochemiluminescence
HR: Heart rate
HC: Hip circumference
SD: Standard deviation
SBP: Systolic blood pressure
METs: Metabolic equivalents
MetS: Metabolic syndrome
NC: Neck circumference
RAS: Renin–angiotensin system
WHR: Waist-to-hip ratio
WC: Waist circumference
VitD: Vitamin D
25(OH)D: 25-hydroxyvitamin D3
